# Slow-paced breathing reduces anxiety and enhances midfrontal alpha asymmetry, buffering responses to aversive visual stimuli

**DOI:** 10.3389/fnhum.2025.1605862

**Published:** 2025-07-14

**Authors:** Tatsuya Iwabe, Akari Miyakawa, Soshi Kodama, Susumu Yoshida

**Affiliations:** ^1^Department of Physical Therapy, School of Rehabilitation Sciences, Health Sciences University of Hokkaido, Tobetsu-cho, Japan; ^2^Department of Physical Therapy, Graduate School of Rehabilitation Science, Health Sciences University of Hokkaido, Tobetsu-cho, Japan; ^3^Department of Occupational Therapy, School of Rehabilitation Sciences, Health Sciences University of Hokkaido, Tobetsu-cho, Japan

**Keywords:** slow-paced breathing, anxiety, frontal alpha asymmetry, aversive visual stimuli, autonomic nervous system activity

## Abstract

**Introduction:**

Slow-paced breathing (SB) reduces anxiety, but its effects on frontal alpha asymmetry (also termed relative left frontal activity, rLFA) and the persistence of these effects after aversive stimuli remain unclear. This study investigated whether SB reduces state anxiety and enhances rLFA, and whether these effects persist immediately after exposure to aversive images from the International Affective Picture System (IAPS) following the breathing task.

**Methods:**

Seventeen healthy participants (7 females) completed sessions of SB (4 s inhalation, 6 s exhalation) and resting breathing (RB). Electroencephalography (EEG), heart rate variability (HRV), respiratory parameters, and State-Trait Anxiety Inventory-State (STAI-S) scores were measured at baseline (pre-task), post-task, and post-stimuli. HRV was evaluated by the root mean square of successive differences (RMSSD) and the low-frequency/high-frequency ratio (LF/HF ratio). Respiratory measurements included respiratory rate, coefficient of variation of respiratory intervals (CVRR), and end-tidal CO_2_ (ETCO_2_). rLFA, measured by alpha wave activity, was calculated at midfrontal (F4-F3) and lateral frontal (F8-F7) EEG sites.

**Results:**

STAI-S scores in SB condition were significantly lower than in RB condition, both post-task (*p* < 0.001, Cohen’s d = −1.46) and post-stimuli (*p* < 0.001, Cohen’s d = −1.25). Midfrontal rLFA (F4-F3) also significantly increased with SB post-task (*p* < 0.01, Cohen’s d = 1.03) and post-stimuli (*p* < 0.05, Cohen’s d = 0.84), whereas lateral frontal rLFA (F8-F7) showed no significant changes. A significant interaction between intervention and time was observed for RMSSD (*p* < 0.01, η^2^G = 0.18). Post-task RMSSD was significantly lower in SB condition compared to RB condition (*p* < 0.001), but this difference was absent post-stimuli.

**Discussion:**

These findings suggest that SB effectively reduces state anxiety while enhancing rLFA, with these effects persisting after exposure to visual stressors. The anxiety-buffering effect of SB may be mediated by enhanced rLFA in the midfrontal region, reflecting improved prefrontal regulatory control over emotion. This indicates that SB could be a practical intervention to enhance neurophysiological resilience against acute stress.

## 1 Introduction

Breathing is an essential autonomic function for sustaining life and a crucial physiological process that bidirectionally interacts with emotion. For example, slow-paced breathing is used to promote mental calmness and has been clinically applied to alleviate anxiety and stress (Brown and Gerbarg, 2005). These breathing interventions reportedly alter autonomic nervous system activity and may modulate anxiety through central nervous system mechanisms (Allen et al., 2023; Folschweiller and Sauer, 2021; Zaccaro et al., 2018). However, despite behavioral and autonomic evidence, cortical-level changes associated with breathing-induced anxiety reduction have not been well characterized.

Frontal alpha asymmetry (FAA) has been investigated as a neurophysiological marker of emotional and cognitive processes. Initially described in relation to approach and avoidance motivation (Coan and Allen, 2004; Davidson, 1992), recent findings suggest that FAA reflects broader regulatory functions (Allen et al., 2018; Harmon-Jones and Gable, 2018; Reznik and Allen, 2018). Also termed relative left frontal activity (rLFA; Reznik and Allen, 2018), FAA is calculated as the log-transformed alpha power difference between homologous frontal sites (log[right] − log[left]); since alpha power inversely reflects cortical activity, higher scores indicate greater left-frontal activation. Elevated rLFA is considered to reflect stronger prefrontal regulatory capacity and reduced vulnerability to stress, whereas diminished rLFA indicates weaker regulatory control and heightened stress reactivity (Allen et al., 2018; Harmon-Jones and Gable, 2018; Mathersul et al., 2008). Despite its theoretical significance, rLFA has rarely been examined in the context of breathing interventions.

Nevertheless, an accumulating body of electrophysiological evidence indicates that respiration modulates cortical α oscillations. A magnetoencephalography study demonstrated that the phase of the respiratory cycle modulates posterior alpha power, and these fluctuations predict near-threshold visual perception (Kluger et al., 2021). Resting-state EEG further demonstrates that alpha phase contains a clear spectral peak at the respiratory frequency, with topographic distributions spanning frontal and occipital regions (Kalauzi et al., 2024). On finer timescales, α power decreases during inspiration and increases during expiration across sensorimotor and bilateral prefrontal cortices (Karjalainen et al., 2025). A brief mindfulness-of-breathing exercise also produces a global α-power elevation persisting into subsequent cognition (Bing-Canar et al., 2016). Because rLFA is defined as the inter-hemispheric difference in frontal α power, any respiration-induced change that is asymmetric—whether phase-locked or amplitude-biased—should translate directly into a shift in rLFA.

Slow-paced breathing reliably attenuates anxiety during and immediately after practice, yet whether this benefit endures once a stressor intervenes remains uncertain. Controlled experiments demonstrate rapid declines in self-reported anxiety and sympathetic activity while participants follow paced-breathing protocols (Arch and Craske, 2006; Zaccaro et al., 2018). Few studies have continued observation into a subsequent challenge, reporting transient reductions in skin-conductance level during painful thermal stimulation and decreases in salivary cortisol after a social-evaluative arithmetic task (Busch et al., 2012; Ma et al., 2017). Critically, none of these investigations measured rLFA, a cortical marker of prefrontal regulation of affect. Clarifying whether rLFA and subjective anxiety remain altered therefore requires sampling both indices immediately after the breathing exercise and again after participants have been exposed to aversive stimuli.

Accordingly, this study aimed to test whether slow-paced breathing modulates state anxiety and rLFA, and whether these effects present immediately after exposure to aversive visual stimuli. We hypothesised that slow-paced breathing would reduce state anxiety and enhance rLFA relative to normal breathing, with these benefits being observable in the period immediately following the stimuli. As an exploratory aim, we examined autonomic and respiratory parameters—heart-rate variability, respiratory rate, respiratory variability, and end-tidal CO₂—to gain a comprehensive view of the underlying physiological mechanisms.

## 2 Methods

### 2.1 Participants

This study included 17 healthy undergraduate and graduate students (7 females, 10 males). The mean age of participants was 22.18 years [standard deviation (SD) = 2.77, range: 20–30 years]. All participants were assessed as being right-handed according to the Edinburgh Handedness Inventory (Oldfield, 1971). A screening questionnaire was used to exclude individuals with a history of smoking, respiratory diseases, diagnosed neurological or psychiatric disorders, receiving current psychiatric treatment, or those unsuitable for electrode attachment due to skin inflammation, injury, or allergies. This study was approved by the Ethics Committee of Health Sciences University of Hokkaido (approval number: 23R1972001) and conducted in accordance with the Declaration of Helsinki. Written informed consent was obtained from all participants.

### 2.2 Psychological and neurophysiological recordings

Subjective anxiety was assessed using the Japanese version of the State–Trait Anxiety Inventory (STAI-JYZ; Hidano et al., 2000). The STAI-JYZ is a 40-item questionnaire with four-point scales measuring state and trait anxiety, with each subscale constituting 20 items. We employed the State Anxiety Assessment of the State–Trait Anxiety Inventory (STAI-S) to measure transient emotional responses, with scores ranging from 20 to 80.

Electroencephalographic (EEG) data were recorded from 32 scalp sites (see Supplementary Figure 1 for the electrode layout) according to the extended International 10–20 system using active Ag/AgCl electrodes (actiCAP, Brain Products GmbH). EEG signals were amplified using a BrainAmp DC amplifier (Brain Products GmbH) and digitized at a sampling rate of 1 kHz. During recording, online filters were applied by the amplifier hardware with a frequency response from 0.016 to 70 Hz. The reference electrode was positioned at FCz and the ground electrode at AFz. Electrode impedances were kept below 10 kΩ. Impedances were checked prior to each session, and signal quality was continuously monitored throughout the recording session. Vertical electrooculograms (EOGs) were simultaneously recorded using bipolar Ag/AgCl electrodes placed above and below the right eye to monitor blink artifacts. The EOG data were subsequently used for artifact rejection during preprocessing.

Heart rate variability (HRV) was derived from electrocardiogram (ECG) recordings. We used a modified Lead II configuration, with Ag/AgCl electrodes (NE-113A, Nihon Kohden) placed on the right clavicle and the left lower rib cage. The ground electrode was placed on the left clavicle. ECG data were sampled at 1 kHz and synchronized with the EEG recordings.

The expired CO_2_ concentration was recorded using a CO_2_ sensor kit (TG-980P, Nihon Kohden) and an end-tidal CO_2_ (ETCO_2_) monitor (OLG-3800, Nihon Kohden). The expired CO_2_ concentration was digitized at 1 kHz and incorporated into the EEG recording system as an analog input for synchronization with EEG data.

### 2.3 Procedure

The experiment was conducted in an electromagnetically shielded room with controlled temperature and humidity. Environmental conditions were maintained at 22–24°C and 40 ± 5% relative humidity to minimize external environmental interference. All experiments were conducted between 13:00 and 17:00 to account for potential physiological variations due to the time of day.

Participants were instructed to abstain from alcohol and caffeine for 12 h before the experiment and to complete their meals at least 2 h before the experiment. Upon arrival at the laboratory, participants completed questionnaires regarding basic attributes (sex, age) and handedness. For physiological measurements, participants were instructed to empty their bladder, after which they were seated in a reclining chair and an experimenter attached the EEG, EOG, and ECG electrodes and the expired CO_2_ sensor.

**Figure 1:**
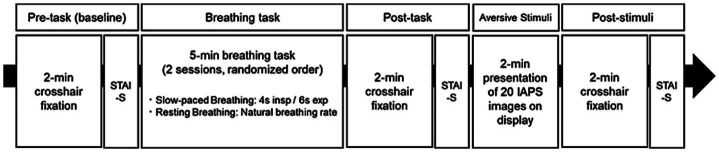
Experimental protocol timeline for investigating the effects of breathing tasks on aversive visual stimuli responses. The experimental protocol constituted two counterbalanced sessions (slow-paced breathing [SB] and resting breathing [RB]), with each session comprising five phases: (1) Pre-task baseline measurement with 2 min crosshair fixation, (2) Breathing task (5 min duration, either 4 s inspiration/6 s expiration for SB or natural breathing rate for RB), (3) Post-task baseline measurement with 2 min crosshair fixation, (4) Aversive visual stimuli presentation (2 min exposure to International Affective Picture System (IAPS) images), and (5) Post-stimuli measurement with 2 min crosshair fixation. The State–Trait Anxiety Inventory-State (STAI-S) was administered after each fixation period. Sessions were separated by a minimum 2 h rest interval, with the order of breathing conditions randomized among participants.

Figure 1 shows the experimental protocol timeline. The experiment comprised two sessions, which were conducted on the same day and separated by a minimum rest period of 2 h. During this rest period, participants remained in a quiet waiting area within the laboratory. To ensure signal consistency, the EEG cap and all other physiological sensors were kept in place throughout the experiment. Measurements were conducted at three time points during each session: before the breathing task (“pre-task” or baseline), immediately after the breathing task (“post-task”), and following exposure to aversive images (“post-stimuli”). Participants completed a 2 min crosshair fixation period at each time point, during which physiological data were recorded, followed by the STAI-S assessment.

The two sessions differed in their breathing task conditions, which were counterbalanced across participants using a randomized block design. Participants in the slow-paced breathing (SB) session followed visual cues to maintain a breathing pattern of 4 s inhalation and 6 s exhalation for 5 min, a duration shown to be sufficient for inducing physiological changes in previous studies (Zaccaro et al., 2018). This pattern with a 0.1 Hz breathing frequency was selected to optimize cardiovascular resonance and vagal activation (Lehrer and Gevirtz, 2014), while ensuring participant comfort. Participants in the resting breathing (RB) session maintained their natural breathing pattern while continuing to fixate on the crosshair. Following the post-task measurements, participants were exposed to aversive visual stimuli.

The aversive stimuli comprised 40 images selected from the International Affective Picture System (IAPS). These images were selected from a subset of 69 IAPS images previously validated to induce anxiety (Costa et al., 2022). Images containing religious elements were excluded based on evaluations by four independent reviewers. The final set of 40 images was divided into two subsets of 20 images, presented randomly in each session. In accordance with the standard protocol for the IAPS (Lang et al., 2008), each image was presented for 6 s without repetition, resulting in a total exposure block of 2 min (120 s). Different subsets were used for each participant to minimize potential order effects from repeated image presentations (Supplementary Table S1). There was no significant difference between these two subsets in normative ratings of valence (t(38) = 0.11, *p* = 0.916) or arousal (t(36.7) = 0.71, *p* = 0.486). Aversive visual stimuli were presented using SuperLab6 (Cedrus) on a 22-inch monitor (1,920 × 1,080 pixels, 60 Hz refresh rate), positioned 1 m from the participant. IAPS images (1,024 × 768 pixels, 4:3 aspect ratio) were presented in the center of the screen, preserving their original resolution and aspect ratio, with dimensions of 21 cm in height and 28 cm in width (approximately 12° vertical and 16° horizontal visual angle).

### 2.4 Data analysis

Prior to preprocessing, all channels were visually inspected for quality. No channels were excluded or required interpolation in this study. The EEG data were analyzed using Brain Vision Analyzer 2.2 (Brain Products GmbH). The EEG data were recorded at 1,000 Hz, re-referenced using the average reference method, and then down sampled to 250 Hz. A zero-phase fourth-order Butterworth bandpass filter (0.1–30 Hz) and a 50 Hz notch filter were applied.

EEG data were segmented into 2-min intervals corresponding to the Pre-task, Post-task, and Post-stimuli crosshair fixation periods. Independent Component Analysis (ICA) was performed on these segmented data to remove eye movement artifacts (Makeig et al., 1997). ICA was applied using the Infomax algorithm (restricted mode). Blink-related components were automatically identified using the Meaned Slope Algorithm based on VEOG activity. Components exceeding a squared correlation threshold of 30% with VEOG signals were removed before back-projection. A maximum of 60 blinks per section (a 2-min recording period) was considered for artifact correction to prevent overcorrection, a threshold based on a plausible physiological blink rate.

The corrected EEG data were further segmented into 2-s epochs with 50% overlap. Epochs with remaining artifacts exceeding ±100 μV were excluded. However, no epochs were excluded after ICA-based artifact correction.

Frontal alpha asymmetry (8–13 Hz) was computed for the midfrontal (F4-F3) and lateral frontal (F8-F7) regions using the natural logarithm transformation: rLFA = ln(right) − ln(left). Power spectral density was estimated using Fast Fourier Transform (FFT) after applying a Hanning window (50% overlap). The employed EEG data processing and analysis procedures were based on established guidelines for frontal EEG asymmetry research (Smith et al., 2017).

HRV analysis was performed using the HRVTool (version 1.07) in MATLAB R2020b (MathWorks) (Vollmer, 2019). The recorded data, originally sampled at 1,000 Hz, were down sampled to 250 Hz. Analysis was conducted on 2 min segments for the Pre-task, post-task, and post-stimuli periods. The root mean square of successive differences (RMSSD) was computed from the R-R interval data as a time-domain measure. Regarding frequency-domain analysis, power spectral density was estimated using FFT to extract low-frequency (LF: 0.04–0.15 Hz) and high-frequency (HF: 0.15–0.40 Hz) components. The LF/HF ratio was calculated as an indicator of sympathetic activity. Prior to analysis, each 2-min R-R interval series was visually inspected for artifacts. Segments containing artifacts, which were observed exclusively at the beginning and end of the recording periods, were excluded from the analysis.

Expired CO₂ waveform data were down sampled to 250 Hz to synchronize with the EEG data and analyzed using custom MATLAB R2020b scripts (MathWorks). A fourth-order Butterworth low-pass filter with a 2 Hz cutoff frequency was applied to reduce noise, followed by zero-phase digital filtering. Moreover, we developed a peak detection algorithm for ETCO₂ detection, with the following parameters: a minimum peak interval of 1.0 s, a minimum peak height equal to the signal mean, and a minimum peak prominence equal to the signal standard deviation. The instantaneous respiratory rate was derived from the time intervals between consecutive ETCO₂ peaks, with values exceeding the median ± 2 standard deviations (SD) removed as outliers. Finally, we computed the mean respiratory rate (RR), the coefficient of variation of respiratory intervals (CVRR), and mean ETCO₂ values from the processed data.

### 2.5 Statistical analysis

Linear mixed models (LMM) were employed to analyze psychological measures (STAI-S) and physiological indices (rLFA at F4-F3 and F8-F7, RMSSD, LF/HF ratio, RR, CVRR, and ETCO_2_). Statistical analyses were conducted using the lme4, lmerTest, emmeans, MuMIn, effectsize, pwr, ggplot2, and gridExtra packages in R (Version 4.4.0).

For each model, the intervention condition (Factor 1: SB, RB) and measurement time point (Factor 2: post-task, post-stimuli) were specified as fixed effects. The corresponding baseline value from the pre-task period was included as a covariate to control for individual baseline differences. We initially attempted to fit models that also included random slopes for the effects of condition and time, but these more complex models failed to converge for some outcomes. Therefore, to ensure model stability and reliable results across all variables, we proceeded with a more parsimonious model that included only random intercepts for participants.

Model fit was evaluated using marginal and conditional R2 values, and residual diagnostics were assessed using QQ plots. Estimated marginal means (EMM) and 95% confidence intervals (CI) were calculated and visualized. Analysis of variance (ANOVA) was conducted using Type III tests with Satterthwaite’s method for denominator degrees-of-freedom to assess the significance of fixed effects in the LMMs.

Statistical significance was set at *p* < 0.05. When a significant interaction was found, simple main effects were analyzed by comparing intervention conditions at each time point and time points within each intervention. If a main effect was significant in the absence of an interaction, *post hoc* pairwise comparisons were conducted on the estimated marginal means. The emmeans package was used for all post hoc tests. Generalized eta squared (η^2^G) was calculated for the ANOVA results to quantify effect sizes, and Cohen’s d was computed for post hoc comparisons. Additionally, post hoc power analyses were conducted based on the observed Cohen’s d values.

## 3 Results

The mean respiratory rate was 6.00 ± 0.11 breaths/min during SB condition and 14.76 ± 3.43 breaths/min during RB condition. These values indicated that participants successfully adhered to the instructed breathing patterns in each condition. The following sections present the results for each physiological and psychological variable. Detailed outputs for the fixed-effects estimates from all linear mixed models are presented in Supplementary Table S2.

### 3.1 State-Trait Anxiety Inventory-State

**Figure 2:**
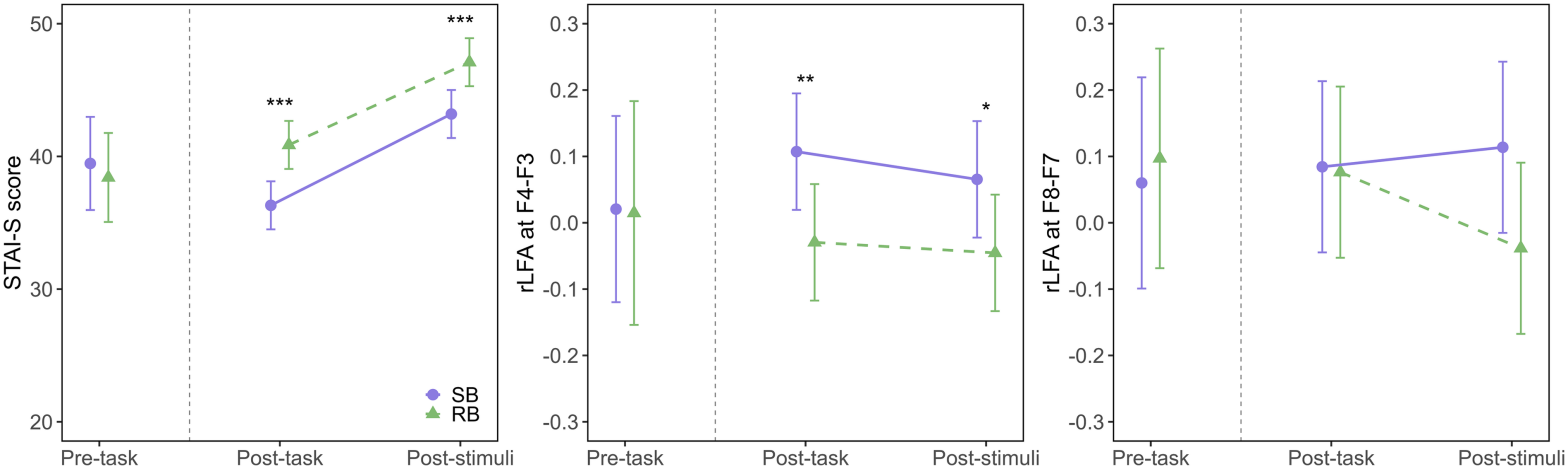
Effects of breathing interventions on state anxiety and relative left frontal activity. Temporal changes in State–Trait Anxiety Inventory-State (STAI-S) scores (left), midfrontal (F4-F3) rLFA (middle), and lateral frontal (F8-F7) rLFA (right) during the experimental protocol. Solid purple lines with circles represent the slow-paced breathing (SB) condition; dashed green lines with triangles represent the resting breathing (RB) condition. Data are presented as estimated marginal means (EMM) with 95% confidence intervals (CI) for post-task and post-stimuli periods, while pre-task data show the raw means with 95% CI. The vertical dashed line separates pre-task from intervention periods. Asterisks indicate significant differences between conditions (*p < 0.05, **p < 0.01, ***p < 0.001).

The model fit evaluation indicated a good fit, with R^2^m = 0.78 and R^2^c = 0.85. The Q-Q plot of standardized residuals did not reveal any significant deviations from normality. The ANOVA results revealed significant main effects of the intervention condition (*F*(1, 47.79) = 30.88, *p* < 0.001, η^2^G = 0.39) and measurement time point (*F*(1, 47.25) = 75.11, *p* < 0.001, η^2^G = 0.61), but no significant interaction between these factors (F(1, 47.25) = 0.18, *p* = 0.671, η^2^G < 0.01). The STAI-S score was significantly lower in SB condition than in RB condition both immediately post-task (SB: EMM = 36.3, 95% CI [34.5, 38.1]; RB: EMM = 40.9, 95% CI [39.1, 42.7], *p* < 0.001, Cohen’s d = −1.46) and after aversive stimuli exposure (SB: EMM = 43.2, 95% CI [41.4, 45.0]; RB: EMM = 47.1, 95% CI [45.3, 48.9], *p* < 0.001, Cohen’s d = −1.25), as shown in Figure 2 (left panel). These results indicated that SB was effective in both reducing state anxiety post-task and attenuating the subsequent anxiety response to the stimuli.

### 3.2 Relative left frontal activity at F4-F3

The model fit evaluation indicated a good fit, with R^2^m = 0.66 and R^2^c = 0.81. The Q-Q plot of standardized residuals did not reveal any significant deviations from normality. The ANOVA results revealed a significant main effect of the intervention condition (*F*(1, 47.72) = 14.84, *p* < 0.001, η^2^G = 0.24), indicating that SB increased rLFA compared to RB. However, the main effect of the measurement time point (*F*(1, 47.70) = 0.80, *p* = 0.374, η^2^G = 0.02) and the interaction (F(1, 47.70) = 0.16, *p* = 0.689, η^2^G < 0.01) were not significant. Post-hoc comparisons showed that the rLFA at F4-F3 was significantly higher in SB condition (EMM = 0.107, 95% CI [0.019, 0.195]) than in RB condition (EMM = −0.030, 95% CI [−0.117, 0.058], *p* = 0.004, Cohen’s d = 1.03) during the post-task period, as illustrated in Figure 2 (middle panel). A similar trend was observed during the post-stimuli period, where rLFA remained significantly higher in SB condition (EMM = 0.065, 95% CI [−0.022, 0.153]) than in RB condition (EMM = −0.045, 95% CI [−0.133, 0.042], *p* = 0.019, Cohen’s d = 0.84).

**Figure 3:**
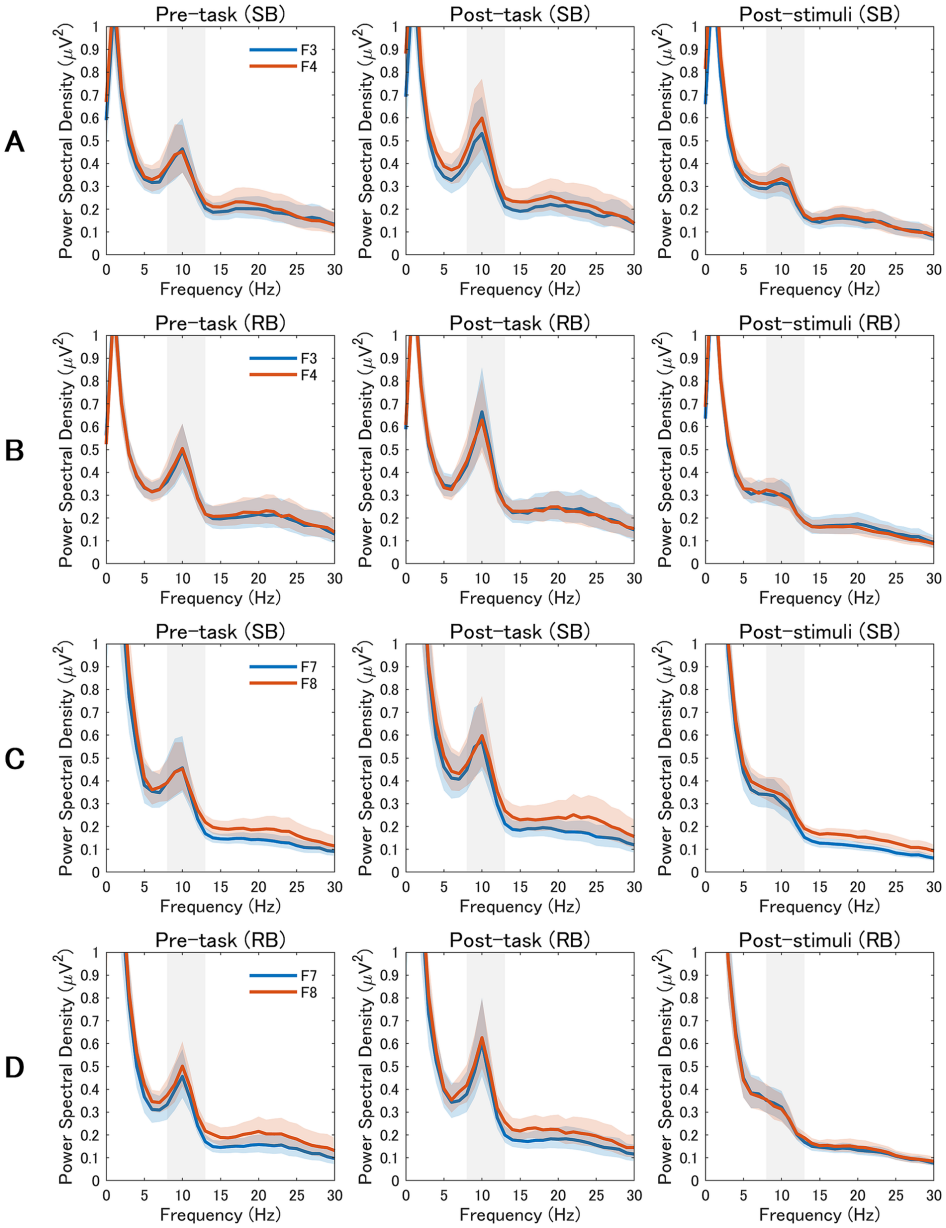
Power Spectral Density Analysis of Frontal and Frontotemporal EEG Activity. EEG power spectra from frontal and frontotemporal electrodes across three experimental periods (pre-task, post-task, and post-stimuli). The alpha band (8–13 Hz) is highlighted in gray. The rows display data from the slow breathing (SB; rows **A,C**) and resting breathing (RB; rows **B,D**) conditions. Rows A and B compare mid-frontal electrodes (F3 vs. F4), while rows C and D compare lateral-frontal electrodes (F7 vs. F8). Blue lines represent left hemisphere electrodes (F3, F7) and orange lines represent right hemisphere electrodes (F4, F8). Shaded areas indicate the standard error of the mean.

As illustrated in Figures 3A,B, power spectral density analysis revealed notable differences in alpha band (8–13 Hz) activity between F3 and F4 electrodes following SB compared to RB. Specifically, in SB condition (Figure 3A), right frontal alpha power (F4) was lower than left frontal alpha power (F3) during both post-task and post-stimuli periods, consistent with the increased rLFA scores. This pattern was less pronounced in RB condition (Figure 3B), reflecting the statistical differences observed in rLFA values between conditions.

### 3.3 Relative left frontal activity at F8-F7

The model fit evaluation showed R^2^m = 0.20 and R^2^c = 0.46. The Q-Q plot of standardized residuals did not reveal any significant deviations from normality. The ANOVA results revealed that the main effects of the intervention condition (*F*(1, 44.54) = 2.29, *p* = 0.138, η^2^G = 0.05) and measurement time point (*F*(1, 44.09) = 0.65, *p* = 0.424, η^2^G = 0.01) were not significant, as shown in Figure 2 (right panel). Additionally, the interaction was not significant (F(1, 44.09) = 1.86, *p* = 0.180, η^2^G = 0.04), indicating that SB did not modulate rLFA at the lateral frontal sites (F8-F7) compared to RB.

Examination of power spectral density patterns in Figures 3C,D confirmed the absence of consistent alpha asymmetry differences between F7 and F8 electrodes across conditions. The spectral profiles for lateral frontal electrodes showed comparable alpha power between left and right hemispheres in both SB and RB conditions, aligning with the non-significant statistical findings for F8-F7 rLFA measures.

### 3.4 Root mean square of successive differences

**Figure 4:**
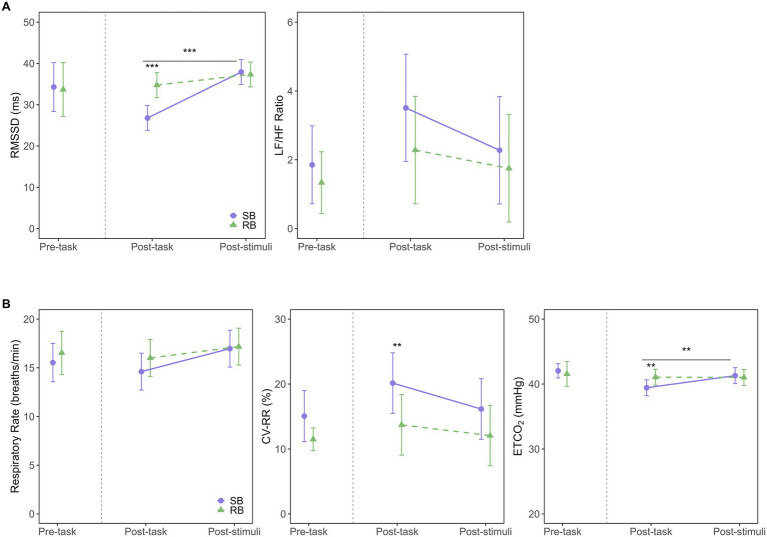
Effects of breathing interventions on autonomic and respiratory parameters. **(A)** Autonomic nervous system responses measured by the root mean square of successive differences (RMSSD; left) and the low-frequency to high-frequency power ratio (LF/HF ratio; right) across experimental periods. **(B)** Respiratory parameters including respiratory rate (left), coefficient of variation of respiratory intervals (CVRR; middle), and end-tidal CO_2_ (ETCO_2_; right) across experimental periods. Solid purple lines with circles represent the slow-paced breathing (SB) condition; dashed green lines with triangles represent the resting breathing (RB) condition. Data are presented as estimated marginal means (EMM) with 95% confidence intervals (CI) for post-task and post-stimuli periods, while pre-task data show raw means with 95% CI. The vertical dashed line separates pre-task from intervention periods. Asterisks indicate significant differences between conditions (**p < 0.01, ***p < 0.001). Horizontal bars with asterisks above them indicate significant differences between time points within the SB condition (**p < 0.01, ***p < 0.001).

The model fit evaluation indicated a good fit, with R^2^m = 0.80 and R^2^c = 0.85. The Q-Q plot of standardized residuals did not reveal any significant deviations from normality. The ANOVA results revealed significant main effects for the intervention condition (*F*(1, 48.01) = 7.96, *p* = 0.007, η^2^G = 0.14) and measurement time point (*F*(1, 47.92) = 27.49, *p* < 0.001, η^2^G = 0.36), as well as a significant interaction (F(1, 47.92) = 10.75, *p* = 0.002, η^2^G = 0.18), as illustrated in Figure 4A (left panel). Post-hoc tests for the interaction showed that in the post-task period, RMSSD was significantly lower in SB condition (EMM = 26.8, 95% CI [23.8, 29.8]) compared to RB condition (EMM = 34.8, 95% CI [31.7, 37.8], *p* < 0.001). Conversely, in the post-stimuli period, there was no significant difference between the conditions (SB: EMM = 37.9, 95% CI [34.9, 40.9]; RB: EMM = 37.3, 95% CI [34.3, 40.3], *p* = 0.749). Analysis of simple main effects within each condition revealed that in SB condition, RMSSD significantly increased from the post-task to the post-stimuli period (*p* < 0.001), while no significant change was observed in RB condition (*p* = 0.171).

### 3.5 Low-frequency/high-frequency ratio

The model fit evaluation showed R^2^m = 0.23 and R^2^c = 0.36. The Q-Q plot of standardized residuals indicated some deviations from normality, with heavy tails observed in the upper range. The ANOVA results revealed that the main effects of the intervention condition (*F*(1, 43.46) = 1.49, *p* = 0.228, η^2^G = 0.03), measurement time point (*F*(1, 41.61) = 1.55, *p* = 0.220, η^2^G = 0.04), and their interaction (F(1, 41.61) = 0.24, *p* = 0.624, η^2^G < 0.01) were not significant. This indicates no statistically significant differences in the LF/HF ratio between SB and RB conditions over time (Figure 4A, right panel).

### 3.6 Respiratory rate

The model fit evaluation showed R^2^m = 0.40 and R^2^c = 0.47. The Q-Q plot of standardized residuals indicated some deviations from normality, with heavy tails observed in the upper range. The ANOVA results revealed that neither the main effect of the intervention condition (*F*(1, 48.59) = 0.80, *p* = 0.375, η^2^G = 0.02) nor the interaction (*F*(1, 47.44) = 0.45, *p* = 0.505, η^2^G < 0.01) reached statistical significance, as shown in Figure 4B (left panel). The main effect for measurement time point approached significance (F(1, 47.44) = 3.97, *p* = 0.052, η^2^G = 0.08).

### 3.7 Coefficient of variation of respiratory intervals

The model fit evaluation showed R^2^m = 0.15 and R^2^c = 0.62. The Q-Q plot of standardized residuals exhibited some departure from normality, with a slight tendency for heavier tails in the upper range. The ANOVA results revealed a significant main effect of the intervention condition (*F*(1, 48.09) = 10.72, *p* = 0.002, η^2^G = 0.18), as shown in Figure 4B (middle panel), indicating that SB significantly increased CVRR compared to RB. However, the main effect of the measurement time point (*F*(1, 47.12) = 3.45, *p* = 0.070, η^2^G = 0.07) and the interaction (F(1, 47.12) = 0.60, *p* = 0.443, η^2^G = 0.01) were not significant. Post-hoc comparisons showed that CVRR was significantly higher in SB condition (EMM = 20.1, 95% CI [15.5, 24.8]) than in RB condition (EMM = 13.7, 95% CI [9.1, 18.4], *p* = 0.005) during the post-task period. This difference was not significant during the post-stimuli period (SB: EMM = 16.2, 95% CI [11.5, 20.8]; RB: EMM = 12.1, 95% CI [7.4, 16.7], *p* = 0.071).

### 3.8 End-tidal CO_2_

The model fit evaluation indicated a good fit, with R^2^m = 0.45 and R^2^c = 0.76. The Q-Q plot of standardized residuals suggested that the data were approximately normally distributed, although slight deviations were observed in the tails. The ANOVA results revealed a significant main effect of the measurement time point (*F*(1, 44.32) = 5.21, *p* = 0.027, η^2^G = 0.11) and a significant interaction (F(1, 44.32) = 5.68, *p* = 0.021, η^2^G = 0.11), as shown in Figure 4B (right panel). Post-hoc tests for the interaction showed that in the post-task period, ETCO_2_ was significantly lower in SB condition (EMM = 39.4, 95% CI [38.2, 40.7]) compared to RB condition (EMM = 41.1, 95% CI [39.8, 42.3], *p* = 0.006). No significant difference was found in the post-stimuli period (*p* = 0.631). Within SB condition, ETCO_2_ levels were significantly lower in the post-task period compared to the post-stimuli period (*p* = 0.002), while no such change was observed in RB condition (*p* = 0.943).

## 4 Discussion

This study examined the effects of SB on state anxiety and rLFA compared to RB and assessed whether these effects persisted immediately after exposure to aversive visual stimuli. The key findings were as follows: (1) SB was significantly more effective than RB in reducing state anxiety post-task, an effect that subsequently buffered against the anxiety increase following aversive stimuli; (2) SB led to a significant increase in rLFA at the midfrontal site (F4-F3) compared to RB, and this effect also persisted immediately after aversive visual stimuli exposure; (3) A significant interaction was observed for RMSSD, an index of parasympathetic nervous system activity. Specifically, during the post-task period, RMSSD was lower in SB condition, but following aversive stimuli, it significantly increased only in SB condition.

The finding that SB buffered against the anxiety response to aversive stimuli is consistent with previous research on the anxiolytic effects of controlled breathing (Brown and Gerbarg, 2005; Jerath et al., 2015; Zaccaro et al., 2018). Our results suggest this buffering is achieved by first lowering state anxiety, which then mitigates the emotional impact of subsequent stressors. This highlights the potential of SB as a proactive strategy for building resilience against acute emotional challenges.

In parallel with the reduction in state anxiety, SB condition produced and maintained a left-lateralized shift in midfrontal rLFA. Although early work framed left-frontal dominance within an approach–withdrawal model of emotion (Coan and Allen, 2004; Davidson, 1992), recent reviews argue that rLFA more broadly indexes prefrontal regulatory control over affect and behaviour (Allen et al., 2018; Reznik and Allen, 2018). From this perspective, the rLFA elevation that persisted across the immediate post-task and post-stimulus intervals likely represents enhanced top-down modulation of subcortical emotion-generating circuits, a mechanism that may underlie the observed attenuation of anxiety in the face of aversive stimuli. This interpretation is strongly supported by cross-sectional evidence indicating that healthy, non-anxious individuals are characterized by greater relative left-frontal activity at rest, whereas individuals with high “anxious arousal” exhibit the opposite pattern of right-frontal lateralization (Mathersul et al., 2008). Thus, the leftward shift induced by SB in our study can be understood as a functional normalization of frontal asymmetry toward a less anxious, more regulated neural state. Accordingly, our findings extend previous work by demonstrating that a brief SB session can recruit and sustain frontal regulatory networks even when participants are exposed to emotionally challenging images.

SB increased left-lateralized rLFA at the midfrontal site (F4–F3) but did not alter the lateral site (F8–F7), indicating regional specificity in prefrontal responsiveness. The midline electrodes overlie medial premotor and dorsomedial prefrontal cortex, nodes of the central autonomic network that receive respiration-synchronous input from brain-stem nuclei and contribute to interoceptive regulation (Folschweiller and Sauer, 2021; Thayer and Lane, 2000). Rodent work indicates that activation of the dorsal anterior cingulate–caudal pontine reticular nucleus (dACC–CPRN) pathway during slow breathing lowers autonomic arousal and reduces anxiety-like behaviour (Jhang et al., 2024), linking respiratory modulation to medial prefrontal activity. The lack of change at F8–F7 may reflect task-related functional demands and recording-related biophysical constraints: lateral prefrontal cortices are primarily engaged by language and executive demands that were not emphasized here, and the associated EEG channels are more vulnerable to temporalis muscle artifacts that can mask subtle alpha-band effects (Smith et al., 2017). Future work should manipulate task demands and optimise signal quality to determine whether slow breathing also influences lateral rLFA.

The neural mechanisms underlying rLFA changes in response to SB remain unclear, but several possibilities can be considered. Previous studies have shown that rLFA is influenced by dopaminergic system activity (Wacker, 2018; Wacker et al., 2013). Therefore, subcortical structures regulated by dopamine may indirectly affect rLFA, considering their central role in processing emotional stimuli and motivation. Notably, studies on rodent suggested that the pathway from the dorsal anterior cingulate cortex to the caudal pontine reticular nucleus is involved in respiratory slowing and attenuation of anxiety-like behaviors (Jhang et al., 2024). Activation of this pathway in mice has been proposed to modulate emotional states through indirect projections to dopamine-associated regions, such as the lateral habenula and the paraventricular thalamic nucleus. Whether similar mechanisms are involved in humans remains unclear; nevertheless, analogous subcortical circuits may have contributed to anxiety reduction and the regulation of anxiety responses to aversive visual stimuli in the present study.

Although slow breathing usually elevates RMSSD by strengthening cardiac vagal activity, we observed a transient reduction during the immediate post-task phase, followed by a marked increase after the stimuli presentation. This pattern can be understood by considering how respiratory sinus arrhythmia (RSA) influences RMSSD. High CVRR values in the post-task window indicated unstable breathing cycles; such variability disperses RSA amplitude across successive beats and can therefore underestimate RMSSD despite genuine vagal activation (Ritz and Dahme, 2006; Schulz et al., 2009). Once breathing regularity returned during the post-stimuli phase, RMSSD rose, revealing the underlying vagal enhancement induced by slow breathing. This temporal dissociation underscores the need to monitor and stabilise breathing patterns when interpreting HRV indices, particularly in individuals with limited practice in paced-breathing techniques (Chinagudi et al., 2014).

ETCO_2_ was significantly lower after SB session, which is consistent with the increased ventilation from deep breathing. A reduction in ETCO_2_ reflects lower arterial CO_2_ levels, which can influence cerebral blood flow and neuronal excitability (Cohen et al., 2002; Seyal et al., 1998; Sparing et al., 2007). This physiological response suggests that the mild hypocapnia induced by SB may have transiently modulated cortical activity (Ozaki and Kurata, 2015). However, ETCO_2_ levels in SB session returned to a level comparable to RB condition after the aversive stimuli; therefore, the effects of CO_2_ were likely temporary and unlikely to have contributed to the sustained changes in state anxiety or rLFA.

This study has several limitations. The lack of pre-registration and *a priori* power analysis suggests that findings on secondary physiological outcomes should be considered exploratory. The small, homogeneous sample of healthy university students limits generalizability, and a larger, more diverse sample is needed to confirm the results. Methodologically, while ICA is a standard procedure for artifact correction, it may have inadvertently removed some neural alpha activity, potentially underestimating alpha power and influencing asymmetry scores. Additionally, future studies could employ more advanced methods like cluster-based permutation tests for a more detailed spectral analysis to complement our primary findings based on rLFA scores. Furthermore, this study’s cross-sectional design does not address the long-term effects of SB, warranting longitudinal studies. Finally, the observed effects were specific to the midfrontal region and to aversive visual stimuli; future research should investigate other cortical areas and a broader range of stressors to enhance ecological validity.

In conclusion, this study provides initial evidence that a brief session of slow-paced breathing can reduce state anxiety and increase relative left frontal activity, with effects persisting immediately after aversive stimuli. These findings highlight the practical potential of SB as an accessible and cost-effective intervention for mitigating anxiety and preventing stress response escalation. However, these promising results must be interpreted with caution due to this study’s limitations, including a small sample size and a cross-sectional design. Therefore, future research is essential to confirm these preliminary findings. In particular, longitudinal studies with larger and more diverse clinical populations are warranted. For instance, neurological disorders such as Parkinson’s disease are frequently accompanied by psychiatric symptoms, including anxiety, which can negatively impact motor function (Avanzino et al., 2018; Harada et al., 2024). Integrating SB into clinical practice for such patients could offer a valuable adjunct to conventional treatments, potentially improving both psychological well-being and motor function and demonstrating the long-term therapeutic benefits of this intervention.

## Data Availability

The original contributions presented in the study are included in the article/Supplementary material, further inquiries can be directed to the corresponding author.
