# Supplementary figures and images for: Slow-paced breathing reduces anxiety and enhances midfrontal alpha asymmetry, buffering responses to aversive visual stimuli
Source: Front Hum Neurosci. 2025 Jul 14;19:1605862. doi: 10.3389/fnhum.2025.1605862 (PMC12301348; doi:10.3389/fnhum.2025.1605862)

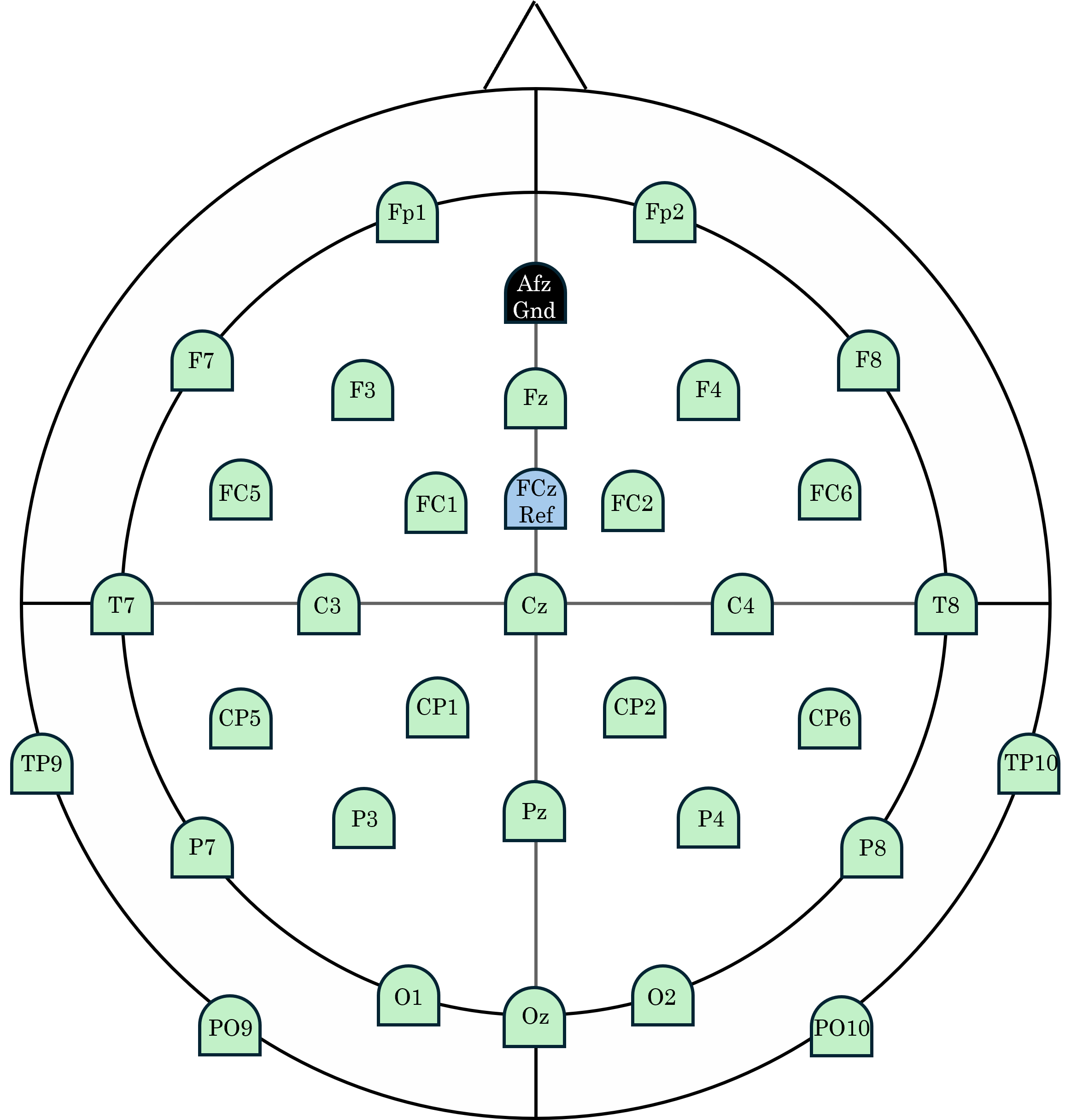

Supplement: Supplementary file 3 [file Image_1.tif]
